# Addition of Berberine to Preservation Solution in an Animal Model of Ex Vivo Liver Transplant Preserves Mitochondrial Function and Bioenergetics from the Damage Induced by Ischemia/Reperfusion

**DOI:** 10.3390/ijms19010284

**Published:** 2018-01-19

**Authors:** Rui Miguel Martins, Anabela Pinto Rolo, João Soeiro Teodoro, Emanuel Furtado, Rui Caetano Oliveira, José Guilherme Tralhão, Carlos Marques Palmeira

**Affiliations:** 1 Department of Surgery, Instituto Português de Oncologia de Coimbra, 3000-075 Coimbra, Portugal; ruirfm@sapo.pt; 2 Department of Life Sciences, Faculty of Sciences and Technology, and Center of Neurosciences and Cell Biology, University of Coimbra, 3004-531 Coimbra, Portugal; anpiro@ci.uc.pt (A.P.R.); jteodoro@ci.uc.pt (J.S.T.); 3 Department of Transplantação Hepática de Crianças e Adultos, Hospitais da Universidade de Coimbra, Centro Hospitalar e Universitário de Coimbra, 3000-075 Coimbra, Portugal; esbfurtado@gmail.com; 4 Department of Pathology, Centro Hospitalar e Universitário de Coimbra, 3000-075 Coimbra, Portugal; ruipedrocoliveira@hotmail.com; 5 Department of Surgery A, Hospitais da Universidade de Coimbra, Centro Hospitalar e Universitário de Coimbra, 3000-075 Coimbra, Portugal; jglrt@hotmail.com; 6 Clínica Universitária de Cirurgia III, Faculty of Medicine, University of Coimbra, 3004-531 Coimbra, Portugal; 7 Center for Investigation on Environment, Genetics and Oncobiology (CIMAGO), Faculty of Medicine, University of Coimbra, 3004-531 Coimbra, Portugal

**Keywords:** liver transplantation, berberine, ischemia/reperfusion injury

## Abstract

Liver transplantation is a therapeutic regimen to treat patients with non-malignant end-stage liver diseases and malignant tumors of hepatic origin. The ischemia/reperfusion (I/R) injury in liver transplantation is associated with disruption of mitochondrial function in the hepatic parenchyma. Several studies have been conducted in animal models to identify pharmacological therapeutic strategies to minimize the injury induced by the cold/warm I/R in liver transplantation. Most of these studies were conducted in unrealistic conditions without the potential to be translated to clinical usage. Berberine (BBR) is a pharmacological compound with a potential protective effect of the mitochondrial function in the context of I/R. For the future clinical application of these pharmacological strategies, it is essential that a close resemblance exists between the methodology used in the animals models and real life. In this study, we have demonstrated that the addition of BBR to the preservation solution in an I/R setting preserves mitochondrial function and bioenergetics, protecting the liver from the deleterious effects caused by I/R. As such, BBR has the potential to be used as a pharmacological therapeutic strategy.

## 1. Introduction

Liver transplantation is an established therapeutic regimen to treat patients with end-stage liver diseases and malignant tumors of hepatic origin. The main limitation of this procedure is related to the shortage of available organs, which has led to the softening of criteria for donor organs (e.g., increased age considered suitable for donors, use of organs after prolonged cold ischemia or donation after cardiac death or hepatic steatosis) [1,2]. This shortage has encouraged the use of organs with lower quality that could compromise clinical outcome [3]. Several animal studies were performed in an attempt to understand the cellular and molecular mechanisms implicated in liver transplantation. One of the major mechanisms involved in this field is the ischemia/reperfusion (I/R) process, in which cellular damage is initiated during hypoxia. This multifactorial process occurs in liver surgery, mostly during liver transplantation, in which there is a period of cold ischemia and warm ischemia followed by a reperfusion period.

The ischemia and reperfusion processes induce cellular injury and the deterioration of mitochondrial function, and the consequent alteration in energy metabolism represents a major cellular event that contributes to hepatic damage.

The decrease in available metabolites and the hypoxic conditions promote the lowering of the mitochondrial respiratory chain activity and ATP synthesis. The reactive oxygen species (ROS) generation increases and promotes the peroxidation of the components of the phospholipids of the inner mitochondrial membrane, as well as of proteins and nucleic acids.

Several therapeutic interventions were performed in animal models (e.g., pharmacological pre-conditioning, drugs, gene targeting strategies, etc.) to mitigate the impact of I/R in the mitochondrial function. Many therapeutic drugs proved themselves to have a protective effect on mitochondrial function, but despite the efforts, few of them were translated into clinical practice. This could be related to the methodology used in some of these studies, which may be unrealistic for clinical usage (i.e., in the clinical setting, it is extremely difficult to infuse drugs in the donor liver several weeks before performing the transplant) [4,5,6,7,8,9,10,11].

One of these promising compounds is berberine (BBR). BBR is a bioactive alkaloid isolated from several plants used in traditional Chinese medicine [12,13]. BBR increases mitochondrial SirT3 activity, which leads to the normalization of the mitochondrial function and also the prevention of liver bioenergetics deterioration caused by the impairing of oxidative phosphorylation [14,15]. Some studies in animal models of liver transplantation showed a mitochondrially-driven protective effect when the animals were pretreated with BBR [16]. Regardless, it is impossible to perform translational research because the methodology used was unfeasible in the clinical setting of liver transplantation. Toward the possible future clinical application of this molecule, we need to understand if the addition of BBR to a liver preservation solution can protect the hepatic cellular and mitochondrial function. Thus, the aim of this study was to evaluate if the addition of BBR to the preservation solution in an I/R setting preserves mitochondrial function.

## 2. Results

### 2.1. Histologic Evaluation

**Figure 1:**
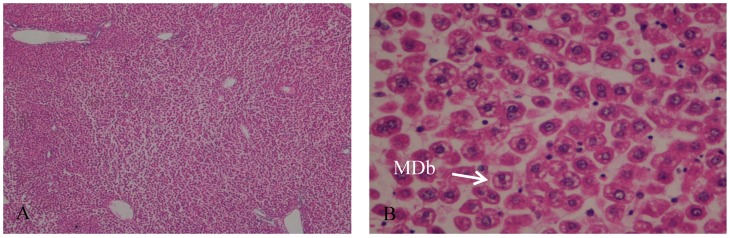
(**A**) Hepatic parenchyma with normal architecture. Moderate to severe disassociation of the hepatocytes, H&E, 40×; (**B**) Hepatocytes preserved the nucleus and intracellular organelles without signals of apoptosis or necrosis. Presence of Mallory-Denk bodies (MDb); H&E, 400×.

**Figure 2:**
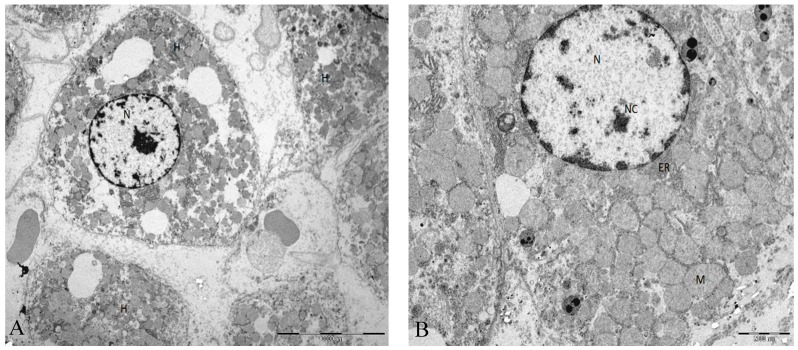
(**A**) Ultrastructural characteristics in rat liver tissue from animals submitted to I/R injury. Moderate to severe disassociation of the hepatocytes. (**B**) The hepatocyte adopts a rounded appearance but with regular contours. Mitochondria are homogeneous, but the main structure is maintained. Abbreviations: H, hepatocyte; N, nucleus; Nc, nucleolus; M, mitochondria; ER, endoplasmic reticulum. Scale bar (**A**): 10000 nm (**B**) 2000 nm.

In the control group, the hepatic tissue maintained the normal liver architecture. In Group B (I/R group), the histologic analysis demonstrates that the hepatic parenchyma also maintains the normal architecture with portal spaces without lesions of their constituents. We observed a moderate to severe disassociation of the hepatocytes, sometimes rounded and with some signals of ballonization, focally with Mallory–Denk bodies (MDb). Hepatocytes preserved the nucleus and intracellular organelles without signals of apoptosis or necrosis (Figure 1 and Figure 2).

**Figure 3:**
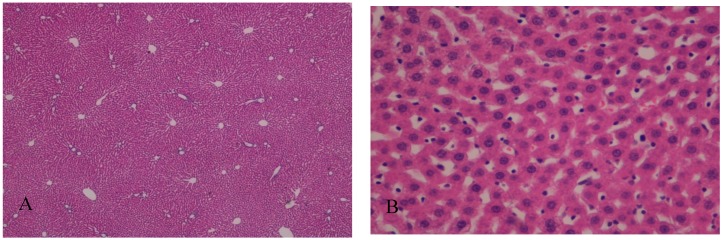
(**A**) The addition of berberine (BBR) in the liver preservation solution preserved the normal liver architecture after I/R injury; H&E, 40×. (**B**) Hepatocytes preserved the nucleus and intracellular organelles. The liver sinusoids present mild pericentrolobular dilation; H&E, 400×.

**Figure 4:**
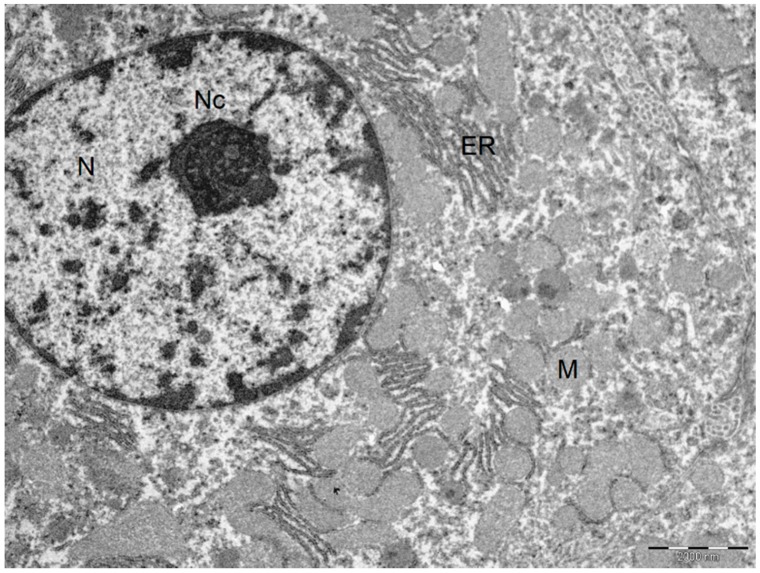
Ultrastructural characteristics with the addition of BBR in the liver preservation solution preserved the normal liver architecture after I/R injury. Normal hepatocyte. Cytoplasm with plasma membranes of adjacent hepatocytes are closely apposed and linear. Mitochondria are homogeneous in size and structure with normal membranes, cristae, matrices and dense calcium-sequestering granules. Abbreviations: N, nucleus; Nc, nucleolus; M, mitochondria; ER, endoplasmic reticulum. Scale bar: 2000 nm

In Group A, with the addition of BBR to the liver preservation solution, the hepatic tissue maintained the usual architecture, without steatosis, inflammatory infiltrate, nor fibrosis. Hepatocytes preserved the nucleus and intracellular organelles without signals of apoptosis or necrosis. The liver sinusoids presented a mild pericentrolobular dilation (Figure 3 and Figure 4).

### 2.2. Mitochondrial Membrane Potential, Δψ

**Figure 5:**
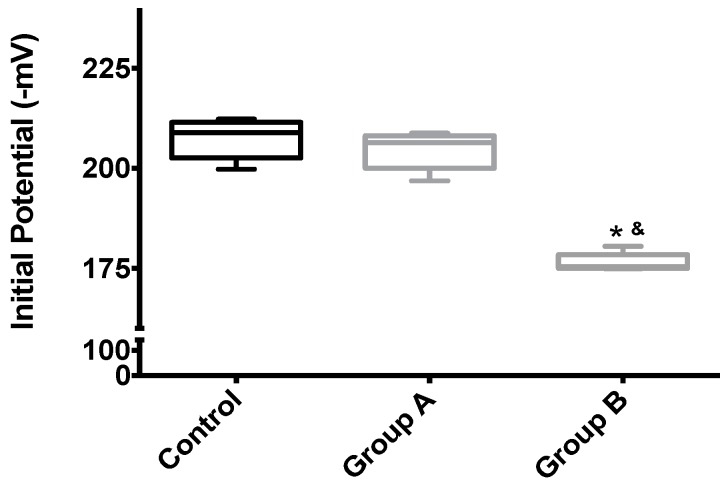
The effects of I/R with or without BBR (18.6 mM) supplementation on hepatic mitochondrial initial membrane potential. Mitochondria were isolated from animals sacrificed after 12 h of hepatic exposure to preservation solution and 1 h of reperfusion, as described in the Materials and Methods Section. Control animals were sham operated. Δψ was evaluated with a TPP^+^-sensitive electrode. Mitochondria isolated from the I/R group (Group B) had a significant loss of membrane potential compared to the control group, while the supplementation with BBR (Group A) was sufficient to prevent said alterations. Data are the means ± 5–95 percentile of at least 2 different experiments, performed with 10 animals/group. * indicates a statistically-significant difference (*p* < 0.05) against the control group; & indicates a statistically-significant difference (*p* < 0.05) against Group A.

**Figure 6:**
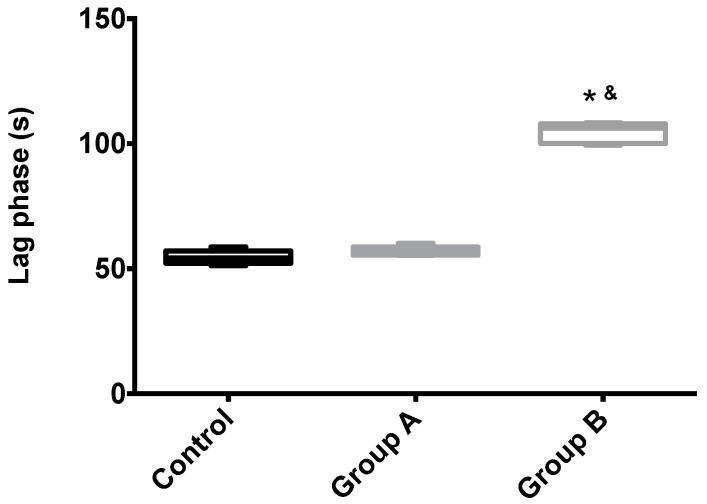
The effects of I/R with or without BBR (18.6 mM) supplementation on hepatic mitochondrial lag phase time. Mitochondria were isolated from animals sacrificed after 12 h of hepatic exposure to preservation solution and 1 h of reperfusion, as described in the Materials and Methods Section. Control animals were sham operated. Lag phase time was evaluated with a TPP^+^-sensitive electrode. Mitochondria isolated from the I/R group (Group B) had a significant increase in the lag phase (required amount of time to phosphorylate the same amount of ADP) when compared to the control group, while the supplementation with BBR (Group A) was sufficient to prevent said alterations. Data are the means ± 5–95 percentile of at least 2 different experiments, performed with 10 animals/group. * indicates a statistically-significant difference (*p* < 0.05) against the control group; & indicates a statistically-significant difference (*p* < 0.05) against Group A.

Mitochondrial membrane potential (Δψ) indicates the phosphorylative capacity of isolated liver mitochondria. In this study, when mitochondria isolated from biopsies were tested using succinate as the energizer, we found a statistically-significant difference in the mitochondrial function parameters between the groups, for all evaluated parameters (initial potential, depolarization, lag phase and repolarization; all *p* < 0.001) (Table 1, Figure 5 and Figure 6). Phosphorylation was induced by the addition of 100 nmol of ADP.

### 2.3. Mitochondrial Respiration

Mitochondrial respiration was quantified by evaluating oxygen consumption after the use of succinate to energize mitochondria. The results obtained are summarized in Table 2. Once again, BBR (Group A) completely preserved mitochondrial function by preventing the alterations caused by I/R (Group B), in all evaluated parameters. Those are State 3 respiration (ADP-stimulated respiration), State 4 respiration (resting state respiration), vFCCP respiration (uncoupled, maximal respiratory activity) and in the indexes RCR (respiratory control ratio, an indicator of coupling and efficiency) and ADP/O (which indicates how many ADP molecules can be phosphorylated per atom of oxygen).

### 2.4. Adenosine Triphosphate Content

**Figure 7:**
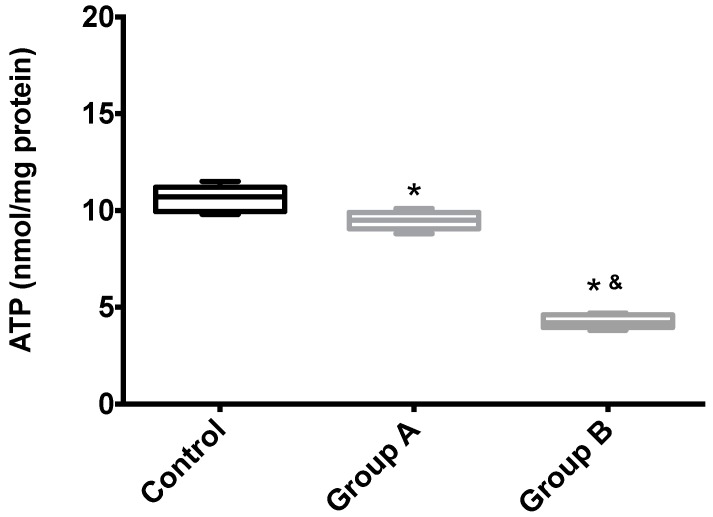
The effects of BBR addition in the preservation solution before ischemia on mitochondrial endogenous ATP content. Mitochondria were isolated from animals sacrificed after 12 h of hepatic exposure to preservation solution and 1 h of reperfusion, as described in the Materials and Methods Section. Control animals were sham operated. ATP content was evaluated with a fluorescent kit, as described in the Materials and Methods Section. As expected, mitochondria isolated from the animals from the I/R livers (Group B) had a significant reduction in endogenous ATP levels when compared with both the control and BBR-supplemented I/R (A) groups. Boxes represent the means ± 5–95 percentile of an *n* = 4 different experiments. * indicates a statistically-significant difference (*p* < 0.05) against the control group; & indicates a statistically-significant difference (*p* < 0.05) against Group A.

Figure 7 shows the hepatic ATP content of Groups A (BBR), B (without BBR) and the control. Unsurprisingly, I/R leads to the almost complete disappearing of ATP from the tissue, an effect that was partially rescued by BRR.

### 2.5. Measurement of the Mitochondrial Permeability Transition

**Figure 8:**
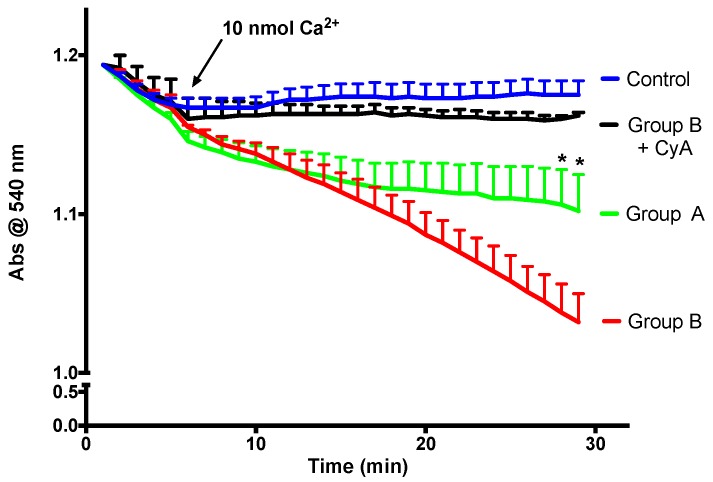
The effect of BBR addition in the preservation solution before ischemia on the susceptibility to the induction of mitochondrial permeability transition (mPT). Mitochondria were isolated from animals sacrificed after 12 h of hepatic exposure to preservation solution and 1 h of reperfusion, as described in the Materials and Methods Section. Control animals were sham operated. Mitochondrial swelling was spectrophotometrically evaluated by light scattering at 540 nm, as described in the Materials and Methods Section. Swelling was induced by the addition of calcium (10 nmol/mg protein). One condition was performed with the previous addition of 1 μM of the mPT inhibitor cyclosporine A (CyA), to exclude any non-mPT-derived swelling. BBR pre-treatment (Group A) significantly reduced the susceptibility of I/R (Group B) mitochondria to calcium-provoked mPT induction. Traces represent the means ± SEM of an *n* = 4 different experiments. A two-way ANOVA with a Sidak post hoc test was conducted to assess the statistical significance between the control and the A and B groups. This test concluded a *p* < 0.05 statistical-significant effect of both time and experimental group. For any given time point, * indicates a *p* < 0.05 difference vs. I/R.

Mitochondrial calcium retention capacity is an important component of mitochondrial cellular activity. After a certain threshold, high molecular weight solutes are released by mitochondria, and this phenomenon is called the mitochondrial permeability transition (mPT). As is visible in Figure 8, in the presence of a relatively high calcium dose and Cyclosporine A (a specific mPT inhibitor), mitochondria do not swell, which is an indicator of the integrity of the isolated mitochondria. There is a direct link between the faster decrease in absorbance and a lower mitochondrial calcium retention capacity. BBR supplementation is able to prevent mPT induction, indicating improvement of calcium retention capacity.

### 2.6. Mitochondrial ROS Generation

**Figure 9:**
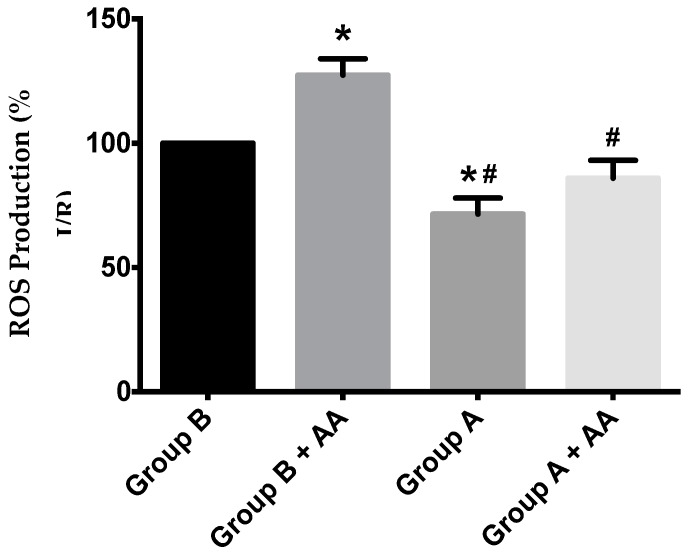
ROS production in hepatic mitochondria of rats subjected to liver ischemia/reperfusion (I/R, Group B), without or with pre-treatment with berberine (BBR, Group A), as evaluated by following 2′,7′-dichlorodihydrofluorescein diacetate (H_2_DCFDA) fluorescence. Mitochondria were isolated from animals sacrificed after 12 h of hepatic exposure to preservation solution and 1 h of reperfusion, as described in the Materials and Methods Section. Control animals were sham operated. After basal H_2_O_2_ production was recorded, antimycin A (AA) 1.25 nmol/mg protein was added to the same wells, and the elevation of the generation of H_2_O_2_ due to the respiratory chain blockade was recorded. I/R (Group B) unexpectedly elevated basal ROS generation over Group A, an effect that was further exacerbated by AA blockade of the respiratory chain activity. BBR supplementation (Group A) reduced ROS generation in both situations back to control levels. Bars represent the means ± SEM of an *n* = 4 different experiments. * indicates a *p* < 0.05 difference vs. Group B; # indicates a *p* < 0.05 difference vs. Group B + AA.

Mitochondrial ROS generation was evaluated by using the H_2_O_2_-sensitive probe, 2′,7′-dichlorodihydrofluorescein diacetate (H_2_DCFDA). Despite a trend towards higher ROS generation in I/R animals, a statistical difference was found between I/R and I/R + BBR (Figure 9).

### 2.7. Western Blotting Analysis

**Figure 10:**
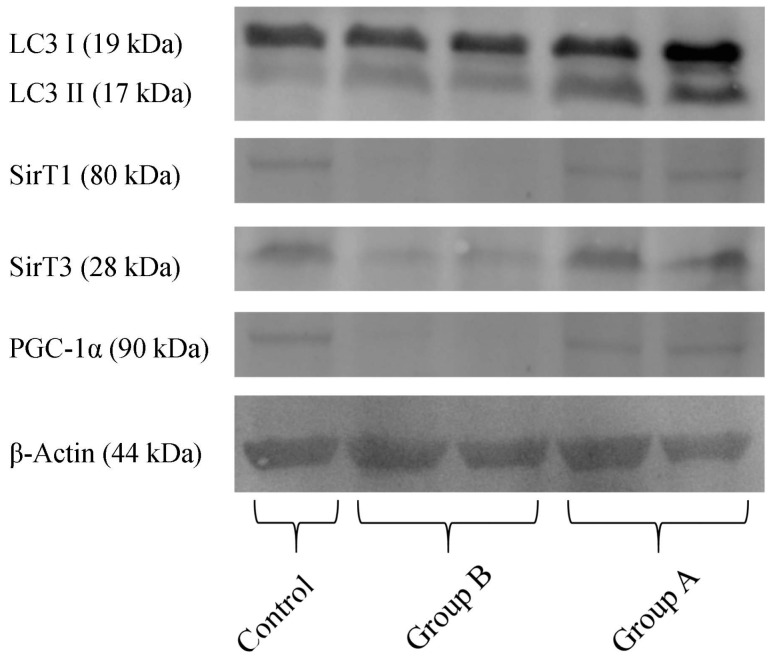
The effect of BBR addition in the preservation solution before ischemia on the content of LC3, Sirt 1 and 3, PGC-1α, as evaluated by Western blot. Tissue samples were obtained from animals sacrificed after 12 h of hepatic exposure to preservation solution and 1 h of reperfusion, as described in the Materials and Methods Section. Control animals were sham operated. I/R livers (Group B) had a reduced content in SirT1, SirT3 and PGC-1α (all these effects were prevented by BBR addition; Group A). The active form of LC3 (LC3 II) was elevated in Group A (BBR addition), when compared to Group B (I/R). The image shows a representative Western blot of these experiments for the respective proteins, from six independent experiments.

Quantification of proteins in the hepatic preparations obtained from the above-mentioned experimental groups (Figure 10) demonstrates that, in BBR pre-treated animals (Group A), there was a higher content in the active form of LC3 (LC3-II), indicating an increase in autophagy, when compared with I/R (Group B). Furthermore, in Group B, there is a severe reduction in the levels of the NAD^+^-dependent deacetylases SirT1 and SirT3, indicating a metabolic compromise in these preparations. BBR pre-treatment restored these proteins’ levels back to control quantities. Finally, regarding the co-activator of transcription (and master regulator of mitochondrial biogenesis) PGC-1α, this protein’s content was highly reduced in I/R preparations, while in BBR pre-treatment, it was restored back to control levels. These data further demonstrate that I/R was severely harmful for hepatic metabolic homeostasis (with particular relevance for mitochondrial function), which was prevented by BBR supplementation. Stimulating autophagy with BBR administration may contribute to eliminating mitochondria damaged during I/R injury, contributing in this way to improved mitochondrial performance.

### 2.8. RNA Isolation and Genetic Expression Evaluation by qPCR

**Figure 11:**
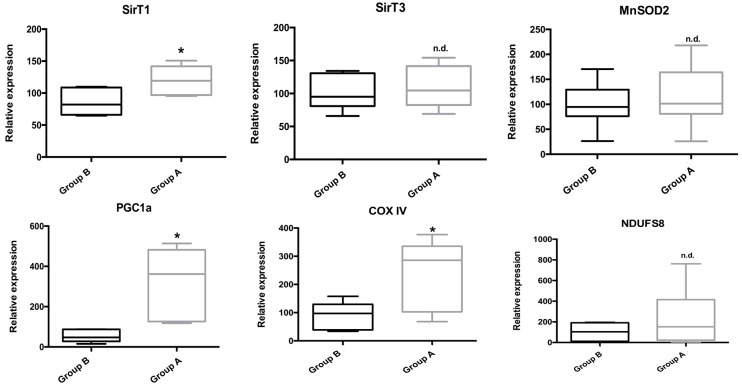
The effect of BBR addition in the preservation solution before ischemia on the gene expression of Sirt 1 and 3, PGC-1α, MnSOD2, COX IV and NDUFS8 as evaluated by qPCR. Tissue samples were obtained from animals sacrificed after 12 h of hepatic exposure to preservation solution and 1 h of reperfusion, as described in the Materials and Methods Section. Control animals were sham operated. I/R livers (Group B) had a reduced gene expression of SirT1, the nuclearly-encoded mitochondrial respiratory complex IV subunit IV (COX IV) and PGC-1α, which were prevented by BBR pre-treatment (Group A). No differences were found in the gene expression of SirT3, the mitochondrial antioxidant protein MnSOD2 and the mitochondrially-encoded mitochondrial respiratory complex I iron-sulfur protein 8 (NDUFS8). Boxes represent the means ± 5–95 percentile of an *n* = 6 different experiments. * indicates a statistically-significant difference (*p* < 0.05) against Group B; n.d., no difference.

Gene expression of several key genes involved in mitochondrial and cellular metabolism, as well as in the inflammatory process was evaluated by real-time PCR (qPCR). As can be seen in Figure 11, BBR addition rescued the expression of genes involved in metabolism (the energy sensor SirT1) and mitochondrial metabolism (PGC-1α, COX IV). Curiously, no gene expression alterations were found for SirT3, MnSOD2 or NDUFS8.

## 3. Discussion

Liver transplantation (LT) has developed during the past few decades from an experimental procedure to the standard of care for patients with end-stage liver disease. The main limitation of this technique is related to the shortage of the liver donor pool and the increase in the number of patients waiting for an organ [17,18,19]. The criteria for organ donation were extended, which has promoted the use of marginal donors previously considered inadequate for LT [20]. This increased the risk of primary graft nonfunctioning after the transplant, which is evidenced in the fatty donor organs where the risk is higher than compared with non-steatotic grafts [21].

I/R injury, especially related to the LT, is a multifactorial process in which cellular damage is induced by a period of hypoxia followed by a one where oxygen is restored. During the reperfusion period, elevated ROS generation causes nonspecific oxidative damage to lipids, proteins and DNA [22,23]. One of the most critical alterations resulting from I/R injury is associated with the deterioration of mitochondrial function and the consequent implication in the energetic metabolism [24]. This rise in ROS generation promotes the peroxidation of the components of the phospholipids of the inner mitochondrial membrane and consequent disruption of the electron flow through the electron transport chain. If this occurs for an extended period, mitochondrial recovery is not possible, and ATP production is permanently decreased [25]. If the mitochondria cannot recover, they are removed through a catabolic pathway of selective autophagy (mitophagy) [26].

BBR, an isoquinoline alkaloid present in several species (e.g., including *Coptis* sp. and *Berberis* sp., among others), has been known in traditional eastern medicine for centuries [27]. BBR has been used for the treatment of several disorders, such as intestinal complications and infections [27,28]. Several studies confirmed that BBR increases antioxidant enzyme activity and glucose uptake while reducing lipid peroxidation [29,30].

The effect of BBR on hepatic injury after liver transplantation is still unclear, despite some reports describing that BBR can ameliorate hepatic injury in liver transplantation by reduction of oxidative stress levels and apoptosis rates, which are involved in the inactivation of mTOR [31]. During I/R injury, hepatocytes activate an autophagy process regulated by several proteins (e.g., Sirt1, BNIP3 and Beclin-1) [32,33]. Our studies showed an important role for SIRT1 and mitochondrial biogenesis in the preventive effects of BBR on diet-induced insulin resistance [15]. Before performing clinical trials, it is important to complete more translational studies in animal models that can be realistically applied in a clinical setting.

In our study, we tried to use a methodology with potential clinical use in a reproducible ex vivo model of I/R, avoiding the complexity and the possible interference in the results related to the high mortality rate of the transplanted animal models [34]. We attempted to closely reproduce in the animal model the reality of the human liver transplantation and tried to understand the influence of adding BBR in the preservation solution, and this relates to mitochondrial function, bioenergetics and the impact in the liver histology.

Regarding the histological changes in the hepatocytes and in the surrounding liver parenchyma, I/R + BBR (Group A) had minimum alterations, as it is comparable to the control group. In these conditions, we identified a normal liver architecture without steatosis, inflammatory infiltrate or fibrosis. The hepatocytes were closely apposed, and the ultrastructural characteristics (nucleus and intracellular organelles) were preserved. On the other hand, without any protective factor, we observed a moderate to severe disassociation of the hepatocytes (I/R group, or Group B). In this condition, hepatocytes became rounder, and while the intracellular organelles were preserved, mitochondria, despite maintaining their main structure, apparently became also rounder. In all the histologic evaluations, there were no signals of apoptosis or necrosis.

We also demonstrate that BBR induces protection against I/R-caused alterations in all evaluated mitochondrial parameters. In fact, the electrochemical potential was maintained at control levels, as were the rates of oxygen consumption in various situations, all of them significantly hampered by I/R. Furthermore, the calcium loading capacity of mitochondria subjected to I/R was severely diminished, an effect partially recovered by BBR supplementation. All these data clearly indicate that, unsurprisingly, the reperfusion period is severely harmful to mitochondrial function, which has been related by our group and others as a key event in the progression of I/R injury. Despite the fact that BBR protective effects of mitochondrial function were already established, we demonstrate that BRR supplementation is a viable strategy to implement in a clinical setting, in order to restore mitochondrial function and thus prevent I/R-related complications. Furthermore, the generation of mitochondrial H_2_O_2_ is increased by I/R, which is undoubtedly related to mitochondrial dysfunction, for the elevation of oxidative stress conditions is a hallmark of I/R injury, leading to a vicious cycle of heightened mitochondrial damage (either by DNA, protein or membrane oxidation and thus injury) and cellular damage, eventually leading to apoptosis and/or necrosis. Once more, BBR supplementation (either by protecting mitochondrial function, or by acting as an antioxidative scavenger, or both) was able to prevent this key phenomenon for I/R injury establishment. All of these parameters indicating mitochondrial dysfunction and cellular stress were further confirmed by the quantification of ATP in hepatic samples from all experimental groups. Recovery from I/R is an energy-exhausting procedure, and an ample supply of ATP is paramount to the recovery of the cell. This, in turn, is highly dependent on the process of mitochondrial oxidative phosphorylation, which conveys over 95% of the cell’s ATP needs. As such, our data on total liver ATP content clearly indicate that I/R leads to the depletion of the liver’s ATP stores, both by the inherences of the I/R injury recovery and by the hampering of mitochondrial function. The latter might be the most important for this question, but not exclusively, since, despite the improvement, BBR pre-treatment did not completely recover hepatic ATP levels. Regardless, there was a massive improvement when compared with the unsupplemented I/R livers, which underscores the important role of mitochondrial function preservation in the context of I/R injury.

To complement our assessment of the effects of BBR on the prevention of I/R-related alterations to hepatic mitochondrial function, gene expression and protein content analyses were performed by means of qPCR and Western blot, respectively. Our data indicate that both gene expression and protein content of the NAD^+^-dependent deacetylase SirT1 were maintained at control levels by BBR supplementation. SirT1 is a key effector in metabolic processes and has been extensively related to both mitochondrial function and cell survival. As such, it appears that BBR might act through the induction of the levels and activity of SirT1. We had previously demonstrated that BBR maintained SirT3 (the mitochondrial equivalent of the cytosolic and nuclear SirT1) activity in the context of high-fat feeding [14], so it is possible that similar mechanisms might be at play here. Curiously, we found no alterations of the gene expression of SirT3, despite a robust effect on protein levels. SirT3 regulates protein acetylation levels within mitochondria and is responsible for the acceleration of the respiratory chain activity upon NAD^+^ levels’ elevation. As such, both proteins’ levels and activity are probably increased as a direct result of the effects of BBR (levels) and the I/R process (which leads to decreased energy and, thus, higher levels of NAD^+^). To the best of our knowledge, no definitive answer regarding the role of BBR on sirtuins’ activity has been shown and as such remains a fascinating avenue of research for future works.

The gene expression and protein levels of the transcription co-activator peroxisome proliferator-activated receptor gamma coactivator 1 alpha (PGC-1α) were also maintained by BBR supplementation. This master regulator of mitochondrial function and biogenesis has been extensively related to increased mitochondrial activity, elevated mtDNA copy number and also to a higher mitochondrial mass. Our data demonstrate that BBR is also important for mitochondrial function by probably elevating the number of mitochondrial units (and thus, mitochondrial respiratory and ATP-generating capacities), through the modulation of PGC-1α levels. This is further confirmed by the elevation of the gene expression of the nuclearly-encoded mitochondrial respiratory complex IV subunit IV (COX IV). Although no alterations were found on the gene expression levels of the mitochondrially-encoded mitochondrial complex I iron-sulfur protein 8 (NDUSF8), or in the protein levels of both and even other mitochondrial respiratory chain complexes’ subunits, it is not possible to rule out mitochondrial biogenesis and elevation of respiratory chain units with our data, since these processes (mtDNA transcription/replication and mitochondrial biogenesis) are complex and time consuming, which given the setting of roughly over 12 h of experimental time, might not be enough to clearly identify those differences. Nevertheless, the indications for the initiation of such processes are here, and given enough time, they might be identifiable (in fact, we expect them to be a key component in BBR-mediated improvement of the outcome of BBR-supplemented I/R surgical events). As such, we propose that BBR might initially act by inducing a quick response of key players (such as sirtuins) while, at the same time, stimulating the processes of a more permanent mitochondrial function maintenance.

Finally, as a clear indicator of the preservation of the cellular and mitochondrial function by BBR in an I/R setting, the levels of active LC3 (LC3-II) protein were found increased. LC3 is an autophagic marker, whose activation leads to the degradation of several cellular components (including dysfunctional mitochondria) as a desperate final attempt by the cell to maintain ATP levels. By stimulating autophagy, BBR is clearly protecting the cell’s energetic levels and thus function, contributing to the observed results and thus to probably maintaining organ viability in a surgical I/R setting.

As such, the improvement in the bioenergetics and mitochondrial function and the preservation of histologic integrity in the liver parenchyma after ischemia/reperfusion confirm the high potential impact of BBR in the field of liver transplantation. More studies are needed to confirm the efficacy and the real value of the addition of BBR in a liver preservation solution and to correlate these findings with biomarkers of the postoperative course in liver transplantation.

## 4. Materials and Methods

### 4.1. Reagents

Except where indicated, all compounds were purchased from Sigma Chemical Co. (St. Louis, MO, USA). All reagents and chemicals used were of the highest commercially available grade of purity.

### 4.2. Animal Study

Twelve-week-old male Wistar rats (*Rattus norvegicus*) weighing 320–350 g were purchased from Charles River (Charles River, Lyon, France). Upon arrival, animals were allowed to acclimatize for one week and were housed in 12 h of light-dark cycles, with controlled temperature and humidity and unlimited access to standard rodent food and acidified water. The study’s protocol was approved by the Animal Ethics Committee of the Faculty of Medicine of the University of Coimbra (ORBEA 150 2016/04112016, 11 April 2016). All studies were conducted in accordance with the principles and procedures outlined as the “3Rs” in the guidelines of the EU (1986/609/EEC and 2010/63/EU), FELASA (Federation of European Laboratory Animal Science Associations) and Animal Research: Reporting of In Vivo Experiments (ARRIVE) and were also approved by the Animal Care Committee of the Center for Neurosciences and Cell Biology, University of Coimbra. We also applied the principles of the ARRIVE guideline for data management and interpretation, and all efforts were made to minimize the number of animals used and their suffering.

### 4.3. Surgical Protocol

The surgical procedures were conducted under anesthesia with ketamine (50 mg/kg) and chlorpromazine (50 mg/kg), provided by the same operator. A median laparotomy was performed, and the liver mobilized by the division of the triangular and hepatogastric ligaments. The experimental model of ex vivo liver transplantation consisted of the introduction of a cannula in the portal vein and hepatic perfusion with a preservation solution (Celsior^®^) at 4 °C for 10 min. After this procedure, we performed a total hepatectomy, taking care to maintain the cannula inside the portal vein. Adequate inflow and outflow were confirmed. Cold static preservation (4 °C) was performed, with or without BBR (18.6 mM) added to preservation solution during 12 h. Reperfusion was performed with Plasma-Lyte 148/Krebs solution (50/50), pH 7.2, supplemented with oxygen during 1 h at 37 °C in a Langendorff system. In the group supplemented with BBR in the preservation solution, the reperfusion also maintained a concentration of BBR of 18.6 mM.

Animals (*n* = 30) were divided into three groups:

Control group (*n* = 10): underwent sham laparotomy, isolation of hepatic pedicle, cannulation of the portal vein, perfusion with a preservation solution at 4 °C for 10 min and total hepatectomy.

Group A (*n* = 10): underwent sham laparotomy, isolation of hepatic pedicle, cannulation of the portal vein; perfusion with a preservation solution at 4 °C for 10 min and total hepatectomy; cold static preservation (4 °C) with BBR (18.6 mM) to the preservation solution during 12 h and reperfusion with Plasma-Lyte/Krebs solution (50/50) with BBR (18.6 mM), pH 7.2, supplemented with oxygen during 1 h at 37 °C.

Group B (*n* = 10): underwent sham laparotomy, isolation of hepatic pedicle, cannulation of the portal vein; perfusion with a preservation solution at 4 °C for 10 min and total hepatectomy; cold static preservation (4 °C) without BBR to the preservation solution during 12 h and reperfusion with Plasma-Lyte/Krebs solution (50/50), pH 7.2, supplemented with oxygen during 1 h at 37 °C.

### 4.4. Mitochondrial Isolation

Mitochondria were isolated in a homogenization medium containing 250 mM sucrose, 10 mM HEPES (pH 7.4), 0.5 mM EGTA and 0.1% fat-free BSA [35,36]. After homogenizing the minced blood-free hepatic tissue, the homogenate was centrifuged at 800× *g* for 10 min at 4 °C. The supernatant was spun at 10,000× *g* for 10 min at 4 °C to pellet mitochondria, which were re-suspended in a final washing medium. EGTA and BSA were omitted from the final washing medium, which was adjusted to pH 7.4. The protein content was determined using the biuret method calibrated with BSA.

### 4.5. Measurement of Mitochondrial Membrane Potential

The mitochondrial membrane potential was estimated using an ion-selective electrode to measure the distribution of tetraphenylphosphonium (TPP^+^). The voltage response of the TPP^+^ electrode to log (TPP^+^) was linear with a slope of 59 ± 1, in conformity with the Nernst equation. Reactions were carried out at 25 °C, in a temperature-controlled water-jacketed chamber with magnetic stirring. Mitochondria (1 mg) were suspended in 1 mL of standard respiratory medium (130 mM sucrose, 50 mM KCl, 5 mM MgCl_2_, 5 mM KH_2_PO_4_, 50 μM EDTA, 5 mM HEPES (pH 7.4) and 2 μM rotenone supplemented with 3 μM (TPP^+^). A matrix volume of 1.1 μL/mg protein was assumed. The measured parameters were membrane potential (mV), depolarization (mV), lag phase (seconds) and repolarization (mV). Readings were recorded in triplicate.

### 4.6. Measurement of Oxygen Consumption

The oxygen consumption of isolated mitochondria was determined by a Clark Type polarographic oxygen electrode (Oxygraph, Hansatech Instruments Ltd., Cambridge, UK) [37]. Mitochondria (1 mg) were suspended under constant stirring, at 25 °C, in 1.4 mL of standard respiratory medium (as above) and 2 μM rotenone. Mitochondria were energized with succinate (5 mM), and State 3 respiration was induced by adding 200 nmol ADP. Oxygen consumption was also evaluated in the presence of 1 μM carbonyl cyanide-*p*-trifluoromethoxyphenylhydrazone (vFCCP). The State 3 respiratory control ratio (RCR) was calculated according to Chance and Williams [38].

### 4.7. Measurement of Adenosine Triphosphate Content

Liver ATP was extracted using an alkaline extraction procedure [39]. Tissue ATP levels were measured with the luciferase/luciferin assay (Sigma Chemical Company, St. Louis, MO, USA) with a PerkinElmer VICTOR 3 plate-reader fluorometer (PerkinElmer, Waltham, MA, USA), according to the manufacturer’s instructions.

### 4.8. Measurement of the Mitochondrial Permeability Transition

Mitochondrial swelling was estimated by changes in light scattering, as monitored spectrophotometrically at 540 nm [40]. Reactions were carried out at 25 °C. Recording was started by the addition of mitochondria (1 mg) to 2 mL of swelling medium (200 mM sucrose, 10 mM Tris-MOPS, 1 mM KH_2_PO_4_ and 10 μM EGTA pH 7.4) supplemented with 3 μM rotenone and 5 mM succinate. After a brief period for the recording of basal absorbance, CaCl_2_ was added, and the resulting alterations in light scattering were registered.

### 4.9. Mitochondrial ROS Generation

ROS generation was fluorometrically determined using a PerkinElmer VICTOR3 plate-reader, with excitation and emission wavelengths of 485 nm of 538 nm, respectively, closely corresponding to the excitation and emission wavelengths of 2′,7′-dichlorodihydrofluorescein diacetate (H_2_DCFDA), a H_2_O_2_-sensitive fluorescent probe [41]. Isolated mitochondria (1 mg /mL) were suspended in standard respiratory medium (as for membrane potential and oxygen consumption recordings) and loaded with succinate 5 mM and H_2_DCFDA 50 μM (prepared in DMSO) for 15 min at 25 °C. After a quick spin-down to remove excess probe and resuspension in 1 mL of fresh, warm respiratory medium, 200 μL of the mitochondrial suspension were loaded into a 96-well plate, and the fluorescence was monitored for 30 min to calculate the rate of ROS formation. The results were expressed as arbitrary relative fluorescence units (RFUs).

### 4.10. Western Blotting Analysis

Tissue homogenates were lysed in ice-cold RIPA lysis buffer supplemented with a cocktail of protease and phosphatase inhibitors (Sigma-Aldrich, St. Louis, MO, USA). Equal amounts of protein (measured with a Bicinchoninic Acid Kit, Sigma-Aldrich) were loaded and electrophoresed on a home-made SDS-polyacrylamide gel and transferred to a polyvinylidene difluoride membrane (Bio-Rad, Hercules, CA, USA). Membranes were blocked with 5% blocking solution (Bio-Rad) for 2 h and incubated in Tris-buffered saline (TBS) supplemented with 1% Tween-20 (TBS-T) and blocking solution 0.5%, overnight at 4 °C, with anti-PGC-1α (Cell Signaling Technology, Beverly, MA, USA, 1:100), anti-SirT1 (Cell Signaling Technology, 1:1000), anti-SirT3 (Cell Signaling Technology, 1:500), anti-LC3 (Sigma-Aldrich, 1:1000) or anti-β-actin (Sigma-Aldrich, 1:5000) antibodies. The following day, membranes were washed at room temperature with TBS–T 3 times for 30 min and incubated with a corresponding secondary antibody (Invitrogen, Carlsbad, CA, USA). Membranes were then washed at room temperature with TBS-T 3 times for 15 min, and immunodetection was performed with the use of the WesternDot 625 goat anti-rabbit or goat anti-mouse Western blot kits (Invitrogen). Membranes were imaged using a VersaDoc Instrument (Bio-Rad).

### 4.11. RNA Isolation and Genetic Expression Evaluation by qPCR

Flash-frozen liver RNA was extracted with an AxyPrep RNA extraction kit (Axygen, Union City, CA, USA). Total RNA was quantified by the use of the Experion Automated Electrophoresis Station (Bio-Rad), and cDNA was generated from RNA with an iScript cDNA synthesis kit (Bio-Rad), following the manufacturer’s recommendations. Semi-quantitative real-time PCR was conducted with a SYBR Green real-time PCR kit (Bio-Rad), following the manufacturer’s recommendations. Utilized primers were, 5′–3′ (*Gene*, Forward primer, Reverse primer): *SirT1*, CCA GAT CCT CAA GCC ATG TT, GAT CCT TTG GAT TCC TGC AA; *SirT3*, ATG GAA AGC TGG ATG GAC AG, CCC TGG TCA GCC TTA ACA AA; *COX IV*, GGC AGA ATG TTG GCT ACC, GCA TAG TCT TCA CTC TTC ACA A; *TNF-α*, ACT CCC AGA AAA GCA AGC AA, CGA GCA GGA ATG AGA AGA GG; *PGC1-α*, CTG CTC TTG AGA ATG GAT ATA CTT, CAT ACT TGC TCT TGG TGG AA; *MnSOD2*, CAC TGT GGC TGA GCT GTT GT, TCC AAG CAA TTC AAG CCT CT; *NDUFS8*, AGT GTA TCT ACT GTG GTT, TAG CTT CTC CTT GTT GTA.

### 4.12. Hematoxylin and Eosin Analysis

Tissue sample analysis was performed on formalin-fixed paraffin-embedded tissue, using a standard procedure: tissue samples were grossly inspected and sectioned, fixed in 4% formaldehyde, embedded in paraffin and cut into 4-μm sections. Examination by an experienced pathologist blinded to the experimental groups was performed on Hematoxylin and Eosin-stained (H&E, Poysciences, Sakura Autostainer-Prisma 81D) slides observed in a light microscope (Nikon Eclipse 50i, Amsterdam, The Netherlands), and images obtained using a Nikon-Digital Sight DS-Fi1 camera.

### 4.13. Transmission Electron Microscopy Analysis

A sample of liver tissue was fixed with 2.5% glutaraldehyde in 0.1 M sodium cacodylate buffer (pH 7.2) for 2 h. Following rinsing in the same buffer, post-fixation was carried out with 1% osmium tetroxide for 1 h. After rinsing in buffer, buffer and distilled water and a final rinsing step in distilled water, 1% aqueous uranyl acetate was added to the samples, for 1 h for contrast enhancement. Following rinsing in distilled water, samples were dehydrated in an ethanol gradient (30–100%), impregnated and embedded in epoxy resin (Fluka Analytical, Seelze, Germany). Ultrathin sections were mounted on copper grids (300 mesh) and stained with lead citrate 0.2%, for 10 min. Observations were carried out on an FEI-Tecnai G2 Spirit Bio Twin at 80 kV.

### 4.14. Data Analysis

All continuous variables were presented as the mean standard ± error of the mean unless otherwise specified. The normality of the distribution was confirmed according to the Kolmogorov–Smirnov and Shapiro–Wilk tests, when indicated. Comparison between groups were performed using Student’s *t*-test, while the differences among ≥3 groups were analyzed by one-way analysis of variance for post hoc multiple comparisons. Statistical analyses were performed using SPSS^TM^ (IBM Corp. (North Castle, NY, USA), Released 2013. IBM SPSS Statistics for Windows, Version 22.0. Armonk, NY, USA: IBM Corp.). Statistical significance was set at *p* < 0.05.

## Figures and Tables

**Table 1:** The effects of I/R with or without BBR (18.6 mM) supplementation on hepatic mitochondrial membrane potential and lag phase. Mitochondria were isolated from animals sacrificed after 12 h of hepatic exposure to preservation solution and 1 h of reperfusion, as described in the Materials and Methods Section. Control animals were sham operated. Δψ was evaluated with a TPP^+^-sensitive electrode. Mitochondria isolated from the I/R group (Group B) had a clear loss of activity compared to the control group, while the supplementation with BBR (Group A) was sufficient to prevent said alterations. Data are the means ± SEM of at least 2 different experiments, performed with 10 animals/group. Statistical significance: *p* < 0.05; ** indicates a statistically-significant difference (*p* < 0.01) against the control group.

	Succinate			
	Control Group	Group A	Group B	*p*-Value
Membrane Potential (−mV)	207.4 ± 5.0	204.5 ± 4.7	176.4 ± 2.3 **	*p* < 0.001
Depolarization (−mV)	24.0 ± 1.0	23.5 ± 1.3	16.9 ± 0.8 **	*p* < 0.001
Repolarization (−mV)	194.7 ± 7.7	197.6 ± 6.5	172.6 ± 2.1 **	*p* < 0.001
Lag Phase (s)	54.6 ± 2.8	57.0 ± 1.9	104.4 ± 4.1 **	*p* < 0.001

**Table 2:** The effects of I/R with or without BBR (18.6 mM) supplementation on hepatic mitochondrial respiration. Mitochondria were isolated from animals sacrificed after 12 h of hepatic exposure to preservation solution and 1 h of reperfusion, as described in the Materials and Methods Section. Control animals were sham operated. Oxygen consumption was polarographically evaluated with a Clark-type oxygen-sensitive electrode. Mitochondria isolated from the I/R group (Group B) had a clear loss of respiratory capacity compared to the group supplemented with BBR (Group A). Data are the means ± SEM of at least 2 different experiments, performed with 10 animals/group. Statistical significance *p* < 0.05; ** indicates a statistically-significant difference (*p* < 0.01) between Groups A and B. RCR, respiratory control ratio.

	Succinate		
	Group A	Group B	*p*-Value
State 3 (natoms O/min/mg protein)	102.8 ± 2.5	65.4 ± 1.4	** *p* < 0.001
State 4 (natoms O/min/mg protein)	17.4 ± 0.6	20.9 ± 0.2	** *p* < 0.001
RCR	5.9 ± 0.3	3.1 ± 0.1	** *p* < 0.001
ADP/O	1.8 ± 0.2	1.48 ± 0.1	** *p* < 0.01
vFCCP (natoms O/min/mg protein)	129.4 ± 3.4	122.0 ± 2.5	** *p* < 0.01
